# Maternal HIV infection and preeclampsia increased risk of low birth weight among newborns delivered at University of Gondar specialized referral hospital, Northwest Ethiopia, 2017

**DOI:** 10.1186/s13052-019-0608-z

**Published:** 2019-01-10

**Authors:** Daniale Tekelia Ekubagewargies, Destaye Guadie Kassie, Wubet Worku Takele

**Affiliations:** 1 Department of Pediatrics and Child Health Nursing, School of Nursing, College of Medicine and Health Sciences, University of Gondar, Gondar, Ethiopia; 2 Department of Community Health Nursing, School of Nursing, College of Medicine and Health Sciences, University of Gondar, Gondar, Ethiopia

**Keywords:** Gondar, Ethiopia, Low birth weight, Newborns

## Abstract

**Introduction:**

Globally, more than 20 million infants are born with low birth weight. The risk of neonatal mortality among low birth weight infants is 25 to 30 times greater than neonates with birth weight **≥** 2500 g. Low birth weight infants are at increased risk of infection, difficulty of feeding, and neurologic problems following birth. So far, the prevalence and factors associated with low birth weight have not been studied in the study area after completion of the time set for millennium development goals. Therefore, the study was aimed at assessing the prevalence and associated factors of low birth weight among newborns delivered at University of Gondar specialized referral hospital, Northwest Ethiopia, 2017.

**Methods:**

Institution-based retrospective cross-sectional study was conducted from April 1 to May 28, 2017. A total of 240 newborns were included in the study. Systematic random sampling technique was used for selecting study participant’s medical record charts from delivery registration log book. Data were collected using data extraction tool. Binary logistic regression followed by multivariable regression model was fitted and interpretation was made based on the adjusted odds ratio and *p*-value of less than 0.05 with corresponding 95% CI.

**Results:**

The prevalence of low birth weight was 12.9% (95%CI: 8.94, 17.83%). No history of preeclampsia (AOR = 0.193, 95%CI: 0.0516, 0.723), negative maternal HIV infection (AOR = 0.015, 95%CI: 0.001, 0.277), and being preterm (AOR = 17.6, 95%CI: 5.18, 60.17) were significantly associated with low birth weight.

**Conclusion and recommendations:**

The result of this study highlighted that the burden of low birth weight is a public health concern among babies delivered in University of Gondar specialized referral Hospital. Maternal HIV infection, preeclampsia, and prematurity were associated with low birth weight. Prevention and treatment of Human immunodeficiency virus infection during pregnancy, tackling prematurity and prevention of preeclampsia through strengthening of antenatal care service and other comprehensive strategies are strongly recommended.

## Background

According to the World Health Organization (WHO), low birth weight (LBW) is defined as weight at birth less than 2500 g [1]. Its severity is categorized as low birth weight (< 2500 g), very low birth weight (< 1500 g), and extremely low birth weight (< 1000 g) [2] [3]. It is one of the major determinants of perinatal survival, developmental disabilities, and illnesses later in life [4]. Globally, it is estimated between 15 and 20% of all births, which is more than 20 million births per year, is LBW [1]. South-central Asia (27%) and sub-Saharan Africa (15%) share the majority of the burden [3]. According to the 2016 Ethiopian Demographic and Health Survey (EDHS) report, about 13% of newborns are LBW [5]. LBW is a significant risk factor for adverse health outcomes including, but are not limited to, respiratory distress syndrome, intracranial hemorrhage, patent ductus arteriosus, necrotizing enterocolitis, and retinopathy of prematurity [6] [7] [8]. Moreover, risk of obesity, metabolic syndrome, neurological impairments, and cardiovascular disease in the later age are observed among LBW babies [9], [10].

It is estimated that LBW contributes to 60 to 80% of all neonatal mortality [6]. A study on the economic burden of LBW showed that it results in lower full-time earning, reduced educational competency, and higher body mass index later in life [11]. LBW newborns stay in hospital on average for 12.9 days after birth, which is far higher as compared to healthy newborns who spend less than 2 days [12]. This is even longer for very low birth weight infants for whom the average hospital stay is 57.5 days [13]. Besides, the average hospital service cost for these babies is 75% higher than babies weighing 1000–1499 g at birth [14].

Studies show that factors contributing to LBW are multifactorial. The following factors have increased the likelihood of being LBW in the newborn; young maternal age, primiparity, lower education level, and poor maternal nutritional status both preconception and during pregnancy [7], [15]. As a matter of fact, empirical studies from different countries show that birth weight outcome is associated with maternal anthropometric measurements [16], [17].

Ethiopia has achieved the fourth Millennium Development Goal (MDG) of the United Nations which targeted to reduce child mortality by two-thirds from 1990 to 2015 [18] [19]. However, there is a scarce of site-specific literature on the magnitude of LBW that shows the progress post-2015 MDG declaration.

Therefore, the aim of this study was to determine the prevalence and factors associated with LBW among newborns delivered at University of Gondar specialized referral hospital. This gives an insight into a useful child health indicator and could help in better evidence-based interventions in Ethiopia aimed at further reducing neonatal mortality.

## Methods

### Study design and setting

A cross-sectional study design was conducted from April 1 to May 28, 2017, at the University of Gondar comprehensive specialized referral Hospital, northwest Ethiopia. The hospital is providing service for more than 7 million people of Gondar town and the catchment area. The maternity division of the hospital is composed of antenatal care (ANC) clinic, postnatal clinic, and two delivery wards. It’s staffed with intern doctors, 37 midwives, 26 obstetricians, and 24 supportive staff. In 2016, a total of 7590 births were attended at the hospital.

### Study participants and eligibility criteria

All weighted live births with complete records delivered at the University of Gondar comprehensive specialized referral Hospital were included.

### Sample size, sampling technique, and sampling procedures

To estimate the sample size, single population proportion formula was used by considering the following statistical assumptions; prevalence of LBW, 17.1% [8], 95% confidence interval (CI), 5% margin of error, and 10% non-response rate to yield a total of 240 study participants. Systematic random sampling technique was employed to select study participant’s medical records. The delivery registration logbook was used as sampling frame. To determine the interval, the number of all births during the study period (648) was divided by the sample size (240) to give 2. To select the first participant among the two, lottery method was used. Accordingly, the second medical record was the starting point to select and every other medical record were included. Whenever the selected chart did not fulfill the criteria (incompleteness), the next medical record was considered.

### Data collection procedure and tools

The patients’ medical records and physician order sheet were used as source of data. The data, consisting of socio-demographic variables, clinical and obstetric history as well as birth outcome, were collected using data extraction tool. Four registered nurses collected the data, while two other supervised the data collection process. Data quality was maintained by the following data quality control mechanisms; the data extraction tool was pretested on 5% of the sample (i.e. 12 charts) at Felegehiwot referral hospital. One day training was given to data collectors and supervisors. Strict supervision of the data collection was carried out throughout the data collection period. The collected data was checked for its consistency and completeness before any attempt to enter, code and analyze it.

### Data processing and analysis

The collected data was coded, entered into Epi-Info version 7, and exported to SPSS version 20 software for analysis. The results were presented using frequency, tables, text, and graphs. Variables with a *p*-value of ≤0.2 in the bi-variable analysis were exported to the multivariable model to control the possible effects of confounders, as well as to identify statistically significant variables. Adjusted odds ratio (AOR) with corresponding 95% confidence interval was computed and variables having *p*-value of less than 0.05 in the multivariable model were considered significantly associated with LBW.

## Results

### Socio-demographic characteristics of participants

A total of 240 participants were enrolled in the study. The mean (+SD) age of the mothers was 27.1 (±5.3) Years. About 59.3% were urban residents and the vast majority (97.8%) of them were married. Moreover, 60.8% of the mothers were unemployed (Table 1).

**Table 1:** Socio-demographic characteristics of study participants at University of Gondar specialized referral hospital, northwest Ethiopia, 2017 (*n* = 240)

Characteristic	Frequency(n)	Percent (%)
Maternal age		
15–19	10	4.2
20–24	68	28.3
25–29	90	37.5
30–34	35	14.6
35–39	37	15.5
40–44		
Residence		
Urban	143	59.6
Rural	97	40.4
Marital status		
Married	233	97.1
Divorced	7	2.9
Occupation		
Unemployed	146	60.8
Governmental	67	27.9
Nongovernmental	27	11.3
Religion		
Orthodox Christian	217	90.4
Muslim	23	9.6

### Medical and obstetric characteristics

One hundred and five (43.5%) of the mothers were primiparous. Almost all (99.8%) of the mothers had 1st ANC follow up. One hundred forty eight (61.9%) had at least four ANC visits during current pregnancy. Dominant number of participants (72.5%) were registered for ANC follow-up in the second trimester. In this study, the majority (86.7%) of pregnant women were supplemented with Iron/Folic acid during the ANC follow up. About one in 10 (10.2%) of participants had history of abortion. Regarding HIV/AIDS infection, the vast majority (97.9%) of the mothers were non-reactive. Majority (85.8%) of women gave birth between 37and 42 weeks of pregnancy. A bit greater than half (54.6%) of newborns were males. Regarding the mode of delivery, 75% were spontaneous vaginal delivery (SVD) (Table 2).

**Table 2:** Medical and Obstetric Characteristics of study participants at Gondar University specialized hospital, 2017. (*n* = 240)

Characteristic	Frequency	Percent
Parity		
1	105	43.8
2	62	25.836
3	36	15.0
4	10	4.2
5	14	5.8
6	9	3.8
Gravidity		
1	98	40.8
2	32	25.8
3	38	15.8
4	14	5.8
5	12	5.0
6	7	2.9
7	7	2.9
8	2	0.8
History of abortion		
Yes	25	10.4
No	215	89.6
Mother Blood group		
A+	83	34.6
A-	2	8
B+	33	13.8
B-	6	2.5
AB	28	11.7
O+	78	32.5
O-	9	3.8
Gestational age by week		
< 37 weeks	24	10.0
37–42 weeks	206	85.8
> 42 weeks	10	4.2
Had ANC follows up		
Yes	239	99.6
No	1	.4
HIV status of the mother		
Negative	235	97.9
Positive	5	2.1
Iron supplement of the mother		
Yes	208	86.7
No	32	13.3
Malaria during pregnancy		
Yes	1	.4
No	239	99.6
TB infection during pregnancy		
Yes	1	.4
No	239	99.6
Had preeclampsia during pregnancy		
Yes	31	12.9
No	209	87.1
Had vaginal bleeding during pregnancy		
Yes	7	2.9
No	233	97.1
Had PROM during pregnancy		
Yes	32	13.3
No	208	86.7
Mode of delivery		
Spontaneous vaginal delivery	180	75.0
Assisted vaginal delivery	15	6.3
Caesarian section	45	18.8
Type of delivery		
Number of ANC follow-up		
One visit	12	5.0
Two visits	37	15.4
Three visits	42	17.5
Four or more visits	148	61.1
Hemoglobin Level of mother		
Anemic (< 12 mg/dl)	40	16.7
Not Anemic (> 12 g/dl)	200	83.3

PROM = Premature Rupture Of Membrane

### Prevalence of low birth weight (LBW)

The prevalence of LBW was 12.9% (95% CI: 8.94, 17.83%). The mean birth weights of newborns was 2882.5 ± 471.3 g. Out of male births, 18 (13.7%) were LBW, while 13 (11.93%) female newborns were LBW.

### Factors associated with LBW

In the bi-variable analysis, history of preeclampsia, hemoglobin level, number of ANC visits, gestational age, maternal HIV infection, place of residence, and iron supplementation were statistically significant at a *p*-value of 0.2. However, in the multivariable model, only gestational age, history of preeclampsia, and maternal HIV infection were significantly associated with LBW.

Accordingly, the likelihood of giving LBW baby among mothers with no history of preeclampsia in the current pregnancy decreased by 81% (AOR = 0.193; [95%CI: 0.0516, 0.723] as compared to their counterparts. The odds of being LBW for babies who were delivered from HIV uninfected mothers was reduced by 98.5% (AOR = 0.015, 95%CI: 0.001, 0.277) as compared to their counterparts. Moreover, mothers who gave birth prematurely (before 37 weeks) were 17.6 times more likely to deliver LBW babies as compared to mothers who gave birth after 37-weeks of gestation (AOR = 17.6, 95%CI: 5.18,60.17). (Table 3).

**Table 3:** Bivariate and multivariate logistic regression analysis of low birth weight at University of Gondar specialized hospital, 2017. (*n* = 240)

Characteristics	Low birth weight		Crude OR	Adjusted OR
	No	Yes		
ANC follow-up visits				
First	7	5	1	1
Second	26	11	0.59(0.15,2.27)	0.67(0.11,4.2)
Third	37	5	**0.19(0.043,0.83)**	0.29(0.039,2.15)
Fourth	139	9	**0.09(0.024,0.34)**	0.16(0.22,1.12)
Preeclampsia				
Yes	23	8	1	1
No	186	23	**0.35(0.14,0.87)**	0.19(0.052,0.722)*
Hemoglobin level				
< 12 mg/dl	27	13	1	1
> 12 mg/dl	182	19	**0.22(0.102,0.53)**	0.30(0.09,1.002)
Gestational age				
< 37 weeks	9	15	**20.83(7.89,54.9)**	17.66(5.18,60.16)*
> 37 weeks	200	16	1	1
Maternal HIV infection				
Reactive	2	3	1	1
Non-reactive	207	28	**0.09(0.014,0.56)**	0.015(0.009,0.28)*
Place of residence				
Urban	130	13	1	1
Rural	79	18	**2.27(1.05,4.90)**	1.39(0.40,4.82)
Iron supplementation				
Yes	189	20	1	1
No	19	12	**5.96(2.53,14.06)**	1.59(0.43,5.88)

*Significant association at a *p*-value of 0.05

## Discussion

Globally, birth weight has been accepted as the single most important determinant of future chances of infant survival, healthy growth and freedom from morbidities and mortalities [4]. The prevalence of low birth weight in this study was 12.9% (95% CI: CI: 8.94, 17.83%). This result suggests that LBW still continues to be a public health problem in the study area. Therefore, a comprehensive strategy has to be designed to combat the problem.

The result of this study is in line with the EDHS report (13%) [6], and other studies conducted in Gondar (11.2%) [20], (Axum 9.9%) [21], and Kenya 12.3% [2].

On the other hand, this study found lower prevalence of LBW as compared to studies conducted in Jimma Zone, Ethiopia (22.5%) [22], Kersa, Ethiopia, (28.3%) [23], and India (20%) [24].The possible reason for this discrepancy might be the time difference in which the studies were conducted. There is almost a decade time gap between the current study and the one conducted in Jimma and Kersa. Advancements in health care coverage as well as quality of care over time could lead to decrement in adverse birth outcomes including LBW. For example, according to the 2016 EDHS report [5], the coverage of ANC service utilization, one indicator of maternal health care delivery, has been improved. With regard to the Indian study, it was community-based study, while the current study is institution-based, more specifically, health institution. Mothers who give birth at health institutions are presumed to receive better ANC follow-up with appropriate interventions that significantly reduces adverse birth outcomes including LBW [8], [23], [25]. There is also half a decade time difference between the current and Indian study.

The result of this study is higher than studies conducted at Laelay Maichew district, Ethiopia (6.3%) [21], Wolaita Sodo, Ethiopia (8.1%) [26], and Yazd, Iran (8.8%) [27]. Differences in living conditions and maternal characteristics may have been reasons for discrepancy as the current study was conducted in urban setting, while the study conducted in Lalay Maichew was in rural setting. The difference in LBW prevalence between the current study and Wolaita Sodo could be due to the fact that the later only considered term deliveries which in fact reduces the prevalence of LBW. The possible reasons for the difference between the current study and a study conducted in Iran could be the difference in socio-demographic characteristics, health service delivery and the way of treating mothers during follow-up care.

The likelihood of giving LBW baby among mothers with no history of preeclampsia in the current pregnancy was decreased by 81% compared to their counterparts. This study is supported by studies conducted in Norway [28], Tanzania [29], and Canada [30]. The possible reason might be due to the reduced placental blood flow that results in decreased fetal growth, with an increased risk of intrauterine growth restriction and low birth weight [31].

The odds of giving birth to LBW baby among mothers who were HIV uninfected was reduced by 98.5% as compared to those mothers who were HIV infected. The finding of this study is in line with a study conducted in Gondar University Hospital [8], Tanzania [29], and a systematic review and meta-analysis study conducted in developing, as well as developed countries [32]. Maternal weight loss secondary to low dietary intake (loss of appetite, mouth ulcers, food insecurity), malabsorption, and altered metabolism is common in HIV infected people [33]. This further causes LBW in newborns as weight is a key maternal anthropometric characteristic that defines birth weight [17]. In utero, HIV infection could also be a risk factor for LBW [34]. In addition, mothers who are on anti-retroviral drugs, especially protease inhibitors are at risk of preterm labor [35], which might in turn cause LBW.

Moreover, this study revealed a positive association between preterm birth and LBW. The likelihood of being LBW baby is increased among premature babies. This is in agreement with studies carried out in Jimma, Ethiopia [22], Gondar, Ethiopia [8], Debrebirhan, Ethiopia [37], Kenya [2], Tanzania [29], and India [36]. This could be due to the fact that preterm birth happens before completion of intrauterine growth, a time when the desired birth weight has not been gained, which leads to LBW [4], [38].

## Conclusion

In summary, this study demonstrated that LBW is a public health problem among babies delivered at the University of Gondar specialized referral Hospital. Absence of maternal HIV infection, preeclampsia, and preterm delivery were factors that positively predicted LBW. Prevention and treatment of HIV infection, prevention of preeclampsia by strengthening ANC services, and preventing prematurity are highly recommended.

## Abbreviations

ANC: Antenatal Care
EDHS: Ethiopian Demographic and Health Survey
HIV: Human Immune Virus
IQR: Inter Quartile Range
LBW: Low Birth Weight
MDG: Millennium Development Goals
WHO: World Health Organization
